# An Energy Efficient MAC Protocol for Multi-Hop Swallowable Body Sensor Networks

**DOI:** 10.3390/s141019457

**Published:** 2014-10-17

**Authors:** Lin Lin, Chengfeng Yang, Kai Juan Wong, Hao Yan, Junwen Shen, Soo Jay Phee

**Affiliations:** 1 School of Mechatronic Engineering and Automation, Shanghai University, Shanghai 200072, China; E-Mails: ycfmaple@163.com (C.Y.); sjwshu@163.com (J.S.); 2 Singapore Institute of Technology, 138683 Singapore; E-Mail: Steven.Wong@SingaporeTech.edu.sg; 3 School of Electronic, Information and Electrical Engineering, Shanghai Jiao Tong University, Shanghai 200240, China; E-Mail: yan_hao@sjtu.edu.cn; 4 School of Mechanical and Aerospace Engineering, Nanyang Technological University, 639798 Singapore; E-Mail: msjphee@ntu.edu.sg

**Keywords:** body sensor networks, MAC, multi-hop, energy efficiency

## Abstract

Swallowable body sensor networks (BSNs) are composed of sensors which are swallowed by patients and send the collected data to the outside coordinator. These sensors are energy constraint and the batteries are difficult to be replaced. The medium access control (MAC) protocol plays an important role in energy management. This paper investigates an energy efficient MAC protocol design for swallowable BSNs. Multi-hop communication is analyzed and proved more energy efficient than single-hop communication within the human body when the circuitry power is low. Based on this result, a centrally controlled time slotting schedule is proposed. The major workload is shifted from the sensors to the coordinator. The coordinator collects the path-loss map and calculates the schedules, including routing, slot assignment and transmission power. Sensor nodes follow the schedules to send data in a multi-hop way. The proposed protocol is compared with the IEEE 802.15.6 protocol in terms of energy consumption. The results show that it is more energy efficient than IEEE 802.15.6 for swallowable BSN scenarios.

## 1. Introduction

**Figure 1.**
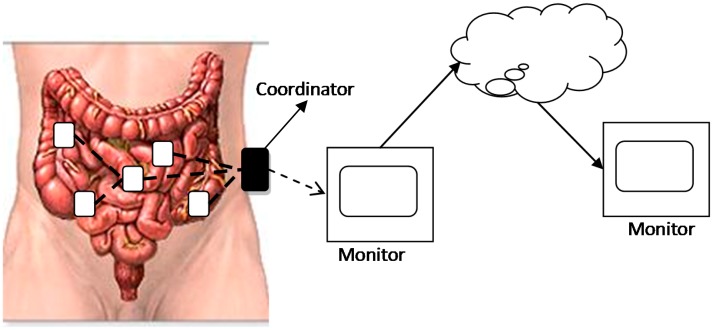
A simple architecture of body sensor networks.

The rapid development of electronic technology and wireless communication enables many kinds of medical sensor devices, which are designed for monitoring the human body. This paper focuses on swallowable medical devices like wireless capsule endoscopes (WCEs) [1]. They are swallowed by patients and go through the gastrointestinal (GI) tract to collect the vital signs of the human body, and send them to a data receiver which is attached onto the abdomen of the human body [2,3]. It can be imagined that more than one medical device can be swallowed and they cooperate to finish some common task, like image segmentation capturing, long term and short term monitoring, *etc.* [4]. In these scenarios, the sensors and the data receiver form a kind of body sensor network (BSN) [5]. Figure 1 shows a simple architecture of this swallowable BSN. Several sensor devices are moving or staying in the GI tract. They collect the sensing data and send them to the coordinator in a single-hop or multi-hop way. The coordinator then transfers the data to a nearby or remote monitor for diagnosis by doctors.

Energy efficiency is the major concern during the design of BSNs. Normally the sensors are powered by batteries and the batteries cannot be replaced if they are inside the human body. The demand for higher resolution and higher sampling frequency of the sensing data makes sensor batteries a big challenge. On the other hand, the path-loss of the body tissue is much larger than through the air, therefore the sensors need larger transmission power to send data. It is well known that the medium access control (MAC) protocol plays an important role in energy management. It can effectively reduce the energy consumption of sensor nodes and prolong the network lifetime. One can focus on the MAC protocol design of BSNs to improve the energy efficiency. The BSN domain can benefit considerably from the appropriate modification and application of modern advances in protocols, techniques and methodologies which have recently been proposed for WSNs pursuing performance improvement in energy efficient routing, QoS, congestion control, congestion avoidance, connectivity and coverage as described in [6–11]. Many energy efficient MAC protocols have already been proposed for WSNs [12–15], however, BSNs have their own attributes which make them different from WSNs [16]. Firstly, WSNs cover a monitoring environment of meters or kilometers, while the scale of BSNs is only as large as the human body (centimeters). The feature makes the coordinator easily reach every sensor node directly. Secondly, WSNs have greater numbers of nodes, ranging from tens to thousands. BSNs have normally less than 10 sensor nodes. Thirdly, BSNs have more energy and processing resource constraints than WSNs. Compared with general BSNs, swallowable BSNs have two main specific features. One is that all the communications are *in-vivo*. The path-loss through body tissue is different from through the air. The other one is that some of the sensor nodes are mobile. The path-loss between sensor nodes varies. The routing may change. It is necessary to consider the above issue and define the specific MAC protocols for specific BSNs.

Several energy efficient MAC protocols for BSNs have been proposed by researchers as surveyed in [17–19]. Alam *et al.*, proposed a traffic-aware dynamic MAC (TAD-MAC) which is considered as an addition in the class of preamble sampling MAC protocols [20]. Every node adapts its wake up interval dynamically with the amount of traffic it receives and consequently optimizes the energy consumption. A traffic status register bank which contains the traffic statistics is used to continuously update the wakeup interval of the receive node with respect to the data transmission rate of transmit nodes. However, this preamble-based protocol is not suitable for BSNs since in most of the applications of BSNs sensors transmit data outside the human body. Preambles cost quite a lot of the energy of the energy-constrained sensors. Marinkovic *et al.* proposed a star topology TDMA-based MAC protocol for remote monitoring of physiological signals [21]. The protocol takes advantage of the static nature of the BSNs to implement an effective TDMA scheme with little overhead. It uses a broadcast network control packet to assign time slots to sensors. The sensors only wake up in their own slots to finish data transmission. This protocol achieves a low duty cycle therefore this reduces the energy consumption. Fang *et al.* proposed BodyMAC which used flexible bandwidth allocation to improve the energy efficiency [22]. Contention free slots are used in the uplink scheme. Different types of bandwidth allocation mechanisms are proposed. Ullah *et al.* proposed a traffic-adaptive MAC protocol, named TaMAC [23]. It dynamically adjusts the duty cycle of the sensor nodes according to their traffic patterns. If a sensor node has no data to send/receive, it will not receive frequent synchronization and control packets. Otal *et al.* integrate a fuzzy-logic system in each body sensor to deal with multiple cross-layer input variables of diverse nature in an independent manner [24]. By being autonomously aware of their current condition, body sensors are able to demand a “collision-free” time slot, whenever they consider it strictly required. Similarly, they may refuse to transmit, if there is a bad channel link, thus permitting another body sensor to do so. The above four proposed protocols do not consider the mobile scenarios. Yoo *et al.* proposed a “pulling” MAC protocol [25]. The coordinator transmits command to ask the data from the sensors. The sensors then send the corresponding data to the base station (BS) in a passive way. There is no need for synchronization. The schedules are controlled by the coordinator. However, the protocol requires sensor nodes to listen all the time without sleep. This will consume quite a lot of energy. In [26], all the activities are also initiated by the master node, but after the link establishment between master node and sensor node, the master node schedules the sleep duration for sensor nodes. Once the sensor wakes up, it listens for the command from the master node. The disadvantage is that there is still a lot of idle listening due to the passive data transfer. An on-demand wakeup radio is proposed to use for BSNs [27]. It allows a device to sleep and be woken up by suitable transmission from another device. This requires a special wakeup circuit in the sensor nodes which increases the complexity. Hyung Tae *et al.* proposed an energy efficient multi-hop communication in body area network [28]. A minimum spanning tree (MST) routing is adopted for the multi-hop network. The coordinator constructs the MST using the battery status of sensor nodes as well as their distances. This proposal does not mention adaptive transmit power which could make the communication more energy efficient and achieve a longer network lifetime. Also due to this, the protocol could not deal well with mobile scenarios. Xiao *et al.* investigated the benefits and limitations of adaptive transmit power control [29]. The adjustment of parameters can achieve different trade-offs between energy saving and reliability, making them suitable across diverse applications under different operation conditions. Li *et al.* proposed a heartbeat driven MAC protocol for BSNs [30]. The heartbeat rhythm information, which is inherent in the human body, is used instead of periodic beacons. Biosensors can extract the heartbeat rhythm from sensing data by detecting waveform peaks. There are two big challenges in terms of energy saving for the protocol implementation. First, the sensors must have extra heartbeat sensors to receive the heartbeat signals. Whether this is really energy and resource efficient is a question. Second, making the rhythm signal acquisition system small and accurate is not easy.

The IEEE 802.15.6 group also released standards for BSNs in 2012 [31]. The MAC protocol adopts a combination of exclusive access period (EAP) and random access period (RAP) using slotted CSMA/CA and managed access period (MAP) which includes scheduled access, unscheduled bi-link access and improvised access. There are three modes for accessing: beacon mode with beacon period superframe boundaries, non-beacon mode with beacon period superframe boundaries and non-beacon mode without beacon period superframe boundaries. The IEEE 802.15.6 standard provides macroscopic and microscopic power management schemes named hibernation and sleep. To hibernate, the sensor nodes can sleep for a number of consecutive beacon periods by setting the wakeup period field in its last connection request frame. Within the beacon frame, a node should wake up to receive and transmit packets in its scheduled allocations. Outside the expected allocations, the node goes into sleep for energy conservation. The main limitation is that the standard does not consider the mobile scenarios. The fixed transmit powers cost energy waste when the sensors are near the coordinator. Another limitation is the ad hoc mode is not clearly defined.

Based on the review and the nature of swallowable BSNs, this paper analyzes the performance of multi-hop communication within the human body and proposes a novel energy efficient solution for medium access control. Compared to other MAC schemes, this paper proposes an uplink, downlink asymmetric network topology. For uplink data transmission, a multi-hop transmission is adopted. In this way the sensor nodes could use less transmission power, therefore saving energy. For downlink data transmission, since the coordinator is outside the human body, normally without energy constraints compared with sensor nodes, single-hop communication is achieved, meaning that the coordinator directly sends data to sensor nodes. This way the sensor nodes do not need to consume extra energy for packet relay and bandwidth is also saved. Since this is a mobile scenario, the path-loss between sensor nodes is dynamic. This paper proposes the adaptive power control scheme trying to optimize the transmit power for energy saving.

The main contributions of this paper are:

1. Evaluation of the performance of multi-hop communication inside the human body in terms of energy consumption based on the IEEE 802.15.6 channel model.
2. Consideration of the mobile scenario of BSNs and proposal of an adaptive power control scheme.
3. Introduction of the dynamic topology of the network. Provision of the TDMA frame and schedules which is a centrally controlled TDMA-based MAC for energy saving. Control messages are transmitted in a single-hop way in both the uplink and downlink direction while the data messages in the uplink direction are transmitted in a multi-hop way.

The rest of the paper is organized as follows: Section 2 discusses multi-hop communications through the human body. Section 3 introduces the details of our energy efficient MAC protocol design. A performance evaluation is given in Section 4. Section 5 concludes the paper.

## 2. Multi-Hop Communications through the Human Body

Multi-hop communication is widely used in WSNs. Short distance radio transmission relay is used instead of a long distance radio transmission, so the transmission power is reduced significantly. In BSNs, the path-loss of the human body is much larger than through the air, so the multi-hop communication is no doubt an interesting research topic for *in-vivo* BSNs. It should also be noted that the energy consumption of a transmitting node is equal to the summation of the transmission energy, which is consumed for the wireless signal transmission from the sender to the receiver, and the circuitry energy, which is consumed by the circuit of the radio and MCU of the transmitting node. Therefore, although the multi-hop communication could reduce the transmission power, the circuitry power consumption would increase due to the packet relay, which cannot be negligible. It is necessary to re-evaluate the performance of multi-hop communication through the human body. This section will examine the energy consumption performance of multi-hop communication via the human body.

An important step in the development of a swallowable body sensor network is the characterization of electromagnetic wave propagation between the devices inside the human body. This paper refers to the IEEE 802.15.6 channel model [32]. The path-loss between any two nodes is both distance and frequency dependent. Equation (1) gives the statistical model of path-loss, where d_0_ is equal to 50 mm as a reference distance, n is the path-loss exponent, S is the variation due to the phenomenon of shadowing and it follows normal distribution with mean of zero and standard deviation of *σ*_s_. The 15.6 Task Group identified seven different propagation scenarios in which the sensor device may operate. For the swallowable BSN applications of this paper, only the scenarios of implant-to-implant and implant-to-body surface are involved. The corresponding parameters are expressed in Table 1. For each scenario, whether the propagation is in the deep tissue or near the body surface is classified. According to this model, the path-loss between any two sensor devices within the human body can be estimated:

(1) $$\begin{array}{c} \text{PL}(\text{d})=\text{PL}({\text{d}}_{0})+10\text{n}\log_{10}\left(\frac{\text{d}}{{\text{d}}_{0}}\right)+\text{S}\\ \text{where S}∼\text{N}(0,\sigma_{\text{S}}) \end{array}$$

The energy model in this section is as follows: For a transmitting node, the total energy consumption (*Pont*) is calculated as two parts: The transmission energy for signal transmission and the circuitry power as shown in Equation (2), where *Pct* is the circuitry power consumption of the transmitter. The signal transmission power consumption (*Pt*) (in dB) is calculated as the sum of path-loss (*PL*) and the receiver sensitivity shown in Equation (3). The power consumption in receive mode (*Ponr*) is mainly composed of receive circuitry power consumption (*Pcr*) as in Equation (4):

(2) Pont=(1+α)×Pt+Pct

(3) Pt=PL+Sensitivity

(4) Ponr=Pcr

In Equation (2), *α* is power amplifier inefficiency factor. It is calculated as in Equation (5), where *β* is the Peak to Average Ratio (PAR) and *μ* is the drain efficiency of the RF power amplifier. *β* is dependent on the modulation scheme and the associated constellation size M in Equation (6). The 4FSK modulation scheme is used by the current commercial WCE and this paper adopts this modulation scheme. Then M is equal to 4, therefore *β* is equal to 1. For *μ* different classes of amplifiers have different values of *μ*. This paper will analyze *μ* ranging from 0.35 to 0.75, which covers most of the range for both linear and non-linear amplifiers. Then, the corresponding *α* ranges from 0.33 to 1.86.

(5) $$\alpha =\frac{\beta }{\mu }-1$$

(6) $$\beta =\frac{3\times \left(\sqrt{M}-1\right)}{\sqrt{M}+1}$$

The circuitry power of the radio is another important parameter. The transmission and receive circuitry power consumptions of the radio have become smaller and smaller as new technologies have been developed. The commercial medical implantable RF transceiver ZL70102 has a TX/RX circuitry power of 5 mA (5 mA × 3 V = 15 mW). Sungho *et al.* presented a 1.5 mA CMOS BFSK transceiver in the MICS band [33]. A 350 μW MSK transmitter and 400 μW OOK receiver for medical implant communications are proposed by Bohorquez *et al.* [34]. Pandey *et al.*, proposed a 90 μW MICS/ISM band transmitter [35]. In this paper, we will evaluate the Tx/Rx circuitry power from 100 μW to 15 mW.

To evaluate the performance of energy consumption of multi-hop communication inside the human body, five scenarios, 1-hop to 5-hop communications, within a fixed distance is simulated. The simulation refers to the IEEE 802.15.6 channel model. The energy model and application traffic are given in Table 2. −84 dBm is the sensitivity of the current commercial wireless capsule. Different circuitry powers from 10 μW to 15 mW are evaluated.

**Figure 2.**
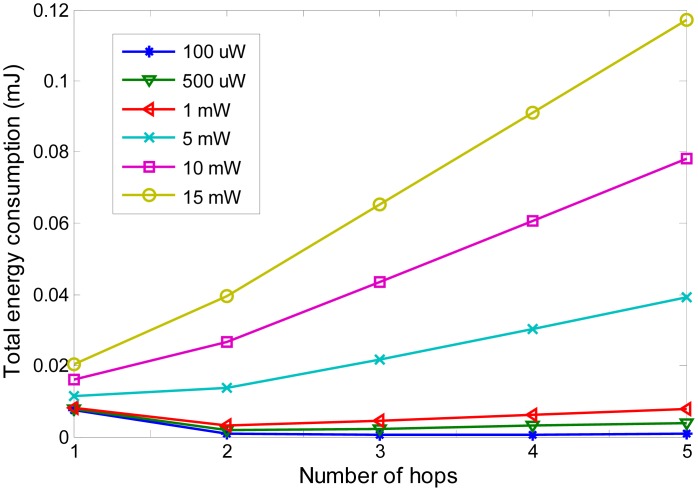
Energy consumption *vs*. number of hops for different circuitry power.

The simulation results are shown in Figure 2. It can be seen that as the number of hops increases, the total energy consumption increases for large circuitry power, while it decreases for small circuitry power. Take 1-hop and 2-hop for example to explain the changes of the energy consumption. The total energy consumption for 1 hop, E_1hop_, is expressed as in Equation (7). Pt1_1hop_ is the transmission power of the first node. T_D_ is the time duration for data transmission. The total energy consumption is composed of two parts: signal transmission energy and circuitry energy. If one relay node is added forming a 2-hop communication, then the total energy consumption, E_2hop_ is expressed as in Equation (8), where Pt1_2hop_ and Pt2_2hop_ represent the transmission power of the first node and the relay node, respectively. From 1 hop to 2-hop, the transmit power reduces because the transmission distance for 2-hop becomes smaller. On the other hand, the relay node consumes energy by receiving and transmitting the data packet. E_1hop_− E_2hop_ is calculated as expressed in Equation (9). Whether 2-hop communication is more energy efficient than 1 hop communication depends on (1 + α) × (Pt1_1hop_ − Pt1_2hop_ − Pt2_2hop_) and (Pct + Pcr). If the former is bigger than the latter, then 2-hop communication saves energy and *vice versa*. The cases for more hops are the same. From Figure 2 it can also be seen that if the circuitry power is bigger than around 3 mW, single-hop wireless communication consumes less energy than multi-hop communication. If the circuitry power drops below 3 mW, multi-hop wireless communication saves power compared with one-hop communication.

(7) $${\text{E}}_{1\text{hop}}=\left((1+\alpha )\times \text{Pt}1_{1\text{hop}}+\text{Pct}\right)\times {\text{T}}_{\text{D}}$$

(8) $${\text{E}}_{2\text{hop}}=\left((1+\alpha )\times \text{Pt}1_{2\text{hop}}+\text{Pct}\right)\times {\text{T}}_{\text{D}}+(\text{Pcr}+(1+\alpha )\times \text{Pt}2_{2\text{hop}}+\text{Pct})\times {\text{T}}_{\text{D}}$$

(9) $${\text{E}}_{1\text{hop}}-{\text{E}}_{2\text{hop}}=\left(\left(1+\alpha )\times (\text{Pt}1_{\text{1hop}}-\text{Pt}1_{\text{2hop}}-\text{Pt}2_{\text{2hop}})-(\text{Pct}+\text{Pcr}\right)\right)\times {\text{T}}_{\text{D}}$$

For 1 mW circuitry power, it can be seen that 2-hop achieve the minimum energy consumption. As explained above, this is because the increase of circuitry power of 2-hop is smaller than the decrease of the transmission power component of 1 hop, and the increase of circuitry power from 2-hop to 3-hop is bigger than the decrease of the transmission power component. The minimum energy consumption shifts from 2-hop to 3-hop, 4-hop when the circuitry power becomes smaller. It is because when the circuitry power becomes smaller, the transmission power becomes dominant.

**Figure 3.**
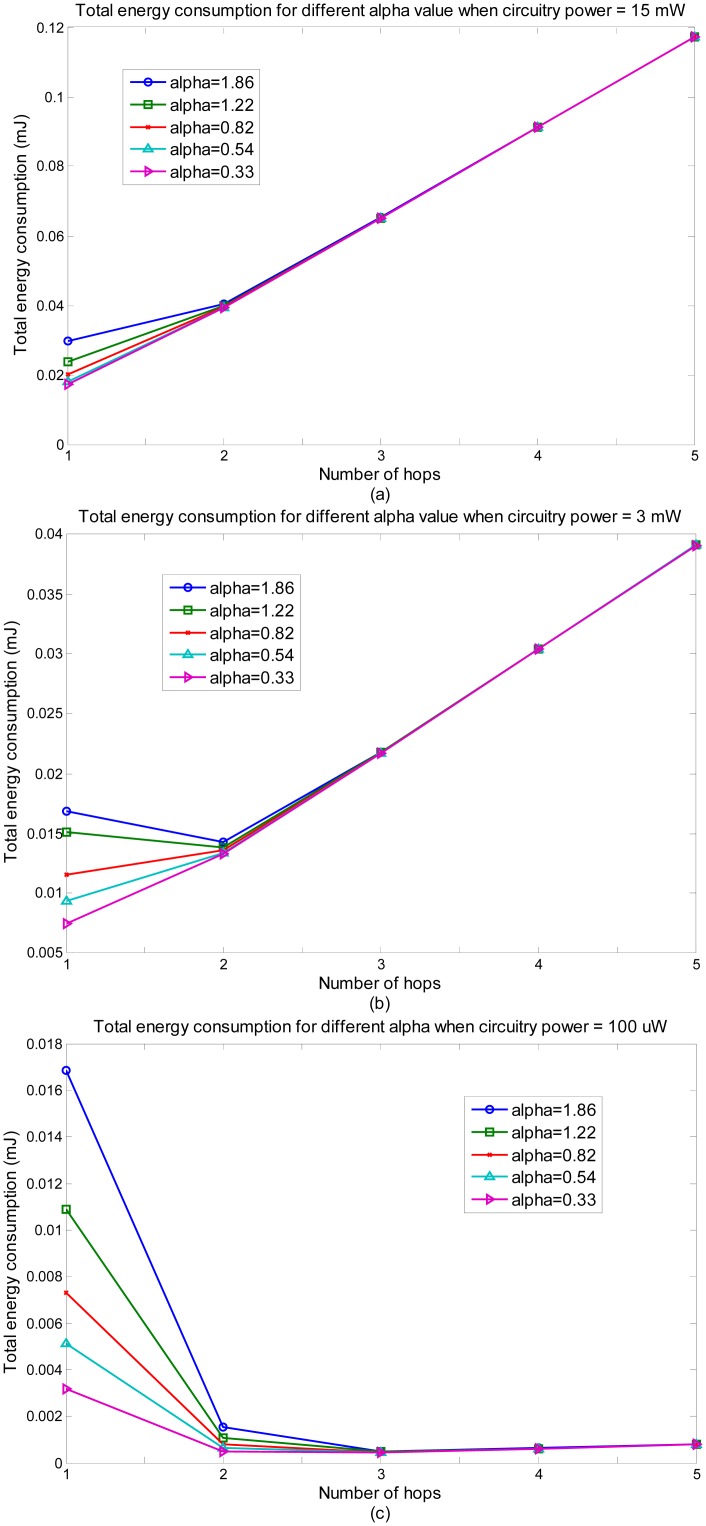
Energy consumption *vs*. number of hops for different α when circuitry power is equal to (**a**) 15 mW; (**b**) 3 mW; (**c**) 100 μW.

Figure 3 shows the influence of α to the total energy consumption with the circuitry power of 15 mW, 3 mW and 100 μW. It can be seen that α influences the total energy consumption significantly for 1 hop and 2 hops. It has little influence for 3 hops and after. The reason is that when 3 hops or above is adopted, the transmission power is very small. According to Equation (3), if Pt is small, then α will have little influence on the whole energy consumption. For 1-hop and 2-hop, Pt is big, so α will have an obvious influence on the whole energy consumption.

For the available chipsets which have a circuitry power of 15 mW, multi-hop communication has no energy saving advantages. When the circuitry power reduces to 3 mW, 2-hop communication can achieve almost the same performance in terms of energy consumption. When the circuitry power reaches 100 μW, which has already achieved in lab, multi-hop communication gives much better performance than single-hop communication for *in-vivo* communication. The reason is the same as the explanation of Figure 2. Given the advancement of electronic technologies, multi-hop communications through the human body will become more and more reasonable and useful.

## 3. Energy Efficient MAC Protocol Design

This section is going to adopt the multi-hop communication in the swallowable BSNs and propose an energy efficient TDMA-based MAC protocol.

### 3.1. Scheduling Design

Since multi-hop communication can save more energy than single hop communication within the human body, it is considered to be used for data transmission. Sensors send data to the coordinator via a multi-hop way. While for downlink data transmission, because the coordinator is not energy constrained, it sends data directly to the sensor nodes with large transmission power. In the swallowable BSN application, because the sensor nodes periodically send sensing data to the coordinator, a TDMA-based MAC scheme is adopted in this paper. Each node only transmits or receives data in the assigned time slot. This scheme can effectively eliminate or reduce collisions, idle listening and overhearing. The stability of the system can also be improved. For the rest of the time slot which does not belong to the sensor node, it will go into the sleep mode for saving energy. The entire scheduling is coordinated by the coordinator which is attached onto the outside of the human body.

**Figure 4.**
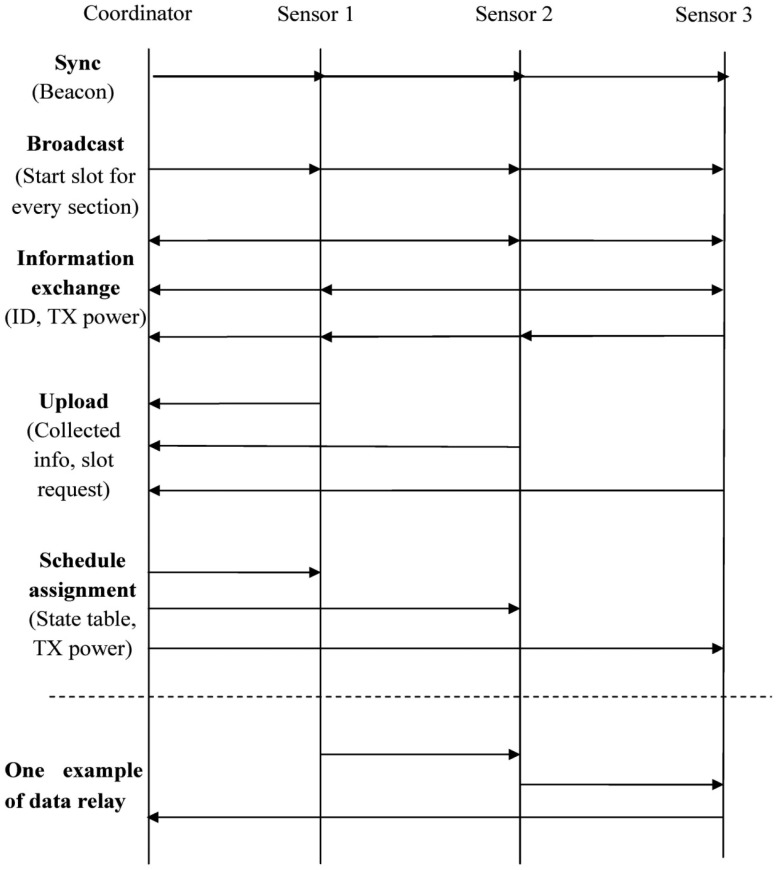
An example of the data transmission schedules in a wireless network with three sensors.

In our protocol, the control messages are exchanged between the coordinator and sensor nodes by direct link. The data messages are sent in the multi-hop way from the sensors to the coordinator. An example of the schedules in a BSN with three sensors is shown in Figure 4. In the beginning the coordinator broadcasts a beacon message to synchronize all the sensor nodes. After that, the coordinator broadcasts a message to announce the starting time slot for the following sections “information exchange”, “neighboring information upload” and “schedule assignment”. All the sensor nodes receive this broadcast information and use their own identification (ID) as the shift to calculate their own transmission time slots for information exchange, neighboring information uploading, and schedule assignment. In the information exchange section, each sensor broadcasts its own information including sensor ID and transmission power in its time slot. In the rest of the time it listens for others' information. In the upload section, the sensors send the collected information and their time slot requests to the coordinator in their own time slots. They go into the sleep status for saving energy when other nodes upload data to the coordinator. After the coordinator receives all this neighboring information, it calculates the routing and slot schedule pattern for each sensor. The time slot assignment is flexible. If a sensor device has a lot of data to send, then it would be given more data slots. If a sensor device has no data to send, then it would not be assigned data slots until next frame. In the schedule assignment section, the coordinator sends the schedules to the sensors. The sensor nodes only receive in their own slots and in the rest of the time they are in the sleep mode. The schedule includes transmit/receive time slot and transmit power for the specific sensor in the data slot section. After that, all sensor devices follow the received schedule to complete the communication. In Figure 4, an example of multi-hop data transmission from sensor 1 to the coordinator via sensor 2 and sensor 3 is shown. The whole process repeats in the next TDMA frame.

**Figure 5.**
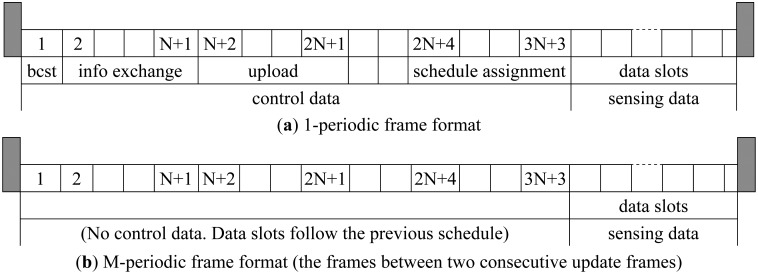
(**a**) 1-periodic TDMA frame format. The frame is composed of broadcast section, power detection section, information upload section, schedule assignment section and data slots section; (**b**) M-periodic TDMA frame format. The frame format of the (*M*−1) frames between two consecutive update frames.

The frame format is shown in Figure 5a. Assume there are *N* sensor nodes in the network. The frame is bounded by the beacon. It is composed of mini control slots and data slots. The mini control slots include broadcast slot (slot 1), information exchange slots (slot 2 to slot *N*+1), neighboring information upload slots (slot *N*+2 to slot 2*N*+1), and scheduling assignment slots (slot 2*N*+4 to slot 3*N*+3). There are two slots (slot 2*N*+2 and slot 2*N*+3) reserved for the coordinator to compute the schedules of sensor nodes. The control data is exchanged using one-hop communication between the coordinator and sensor nodes, which means the sensor nodes need to use a larger transmission power to send the control data to the coordinator, but for the sensing data transmission, the sensor nodes use little transmission power to send data to the coordinator in a multi-hop way.

**Figure 6.**
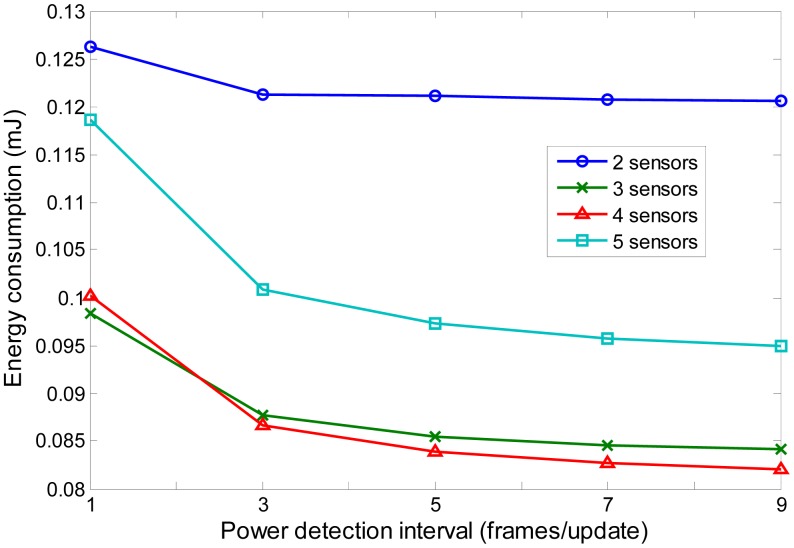
Energy consumption *vs*. information exchange interval. 1 frame/update represents 1-periodic update. 3–9 frames/update represent an M-periodic update.

Since the sensor nodes moves rather slowly (0.2 mm/s) in the human body, there is no need to conduct information exchange and update the routing and transmission power for every frame. Therefore, M-periodic update is proposed for energy saving. In the proposed protocol, the information exchange and routing update are conducted in an M-periodic way (The information exchange and routing update is conducted every M frame). The update frame is the same as 1-periodic frame in Figure 5a. The (*M*-1) frames between two consecutive update frames contain only data slots as shown in Figure 5b. They follow the same schedules in the data slots as in previous update frame. The sensor nodes go into sleep mode in the mini control data slots during these (*M*-1) frames. When the next update frame comes, the sensor nodes as well as the coordinator repeat the same schedules. Figure 6 compares M-periodic and 1-periodic information exchange for different network sizes. The setup is the same as in Section 2. For x-axis, 1 frame/update represents 1-periodic update. 3–9 frames/update represents M-periodic update. It can be seen that the total energy consumption reduces when the update interval increases. As the update interval increases, the energy consumption remains flat. This is because the control packet is already very small compared with the sensing data. It is also noted that 3-sensor and 4 sensor networks achieve lower energy consumption than 2 sensor and 5 sensor networks. This matches the result in Figure 2. The minimum energy consumption is obtained for certain hop. If the hop number increases, the energy consumption will increase.

### 3.2. Routing and Transmit Power Calculation

The coordinator collects the neighboring information from the sensor devices and calculates the schedules including the routing, transmission power and the slot assignment. The path-loss, *PL*, between any two sensors is calculated using the transmit power and received signal strength indication (*RSSI*) Equation (10). *Pt* is the transmission power from node *i* to node *j*. *RSSI* is the receiving power of node *j*. To calculate the routing for each sensor, the coordinator firstly initializes the next hop array and total power array. The initial value of next hop ID is pointed to the coordinator. The total power for data transmission from node *i* to node *j* is calculated as the power consumption for data transmission from node *i* to the coordinator calculated according to Equation (11). Σ_route_*Pt_ij_* is the summation of transmission power in the route. Σ_route_*Pr_ij_* is the summation of receive power except the coordinator. Excluding the receive power consumption of the coordinator is because the coordinator is outside the human body with large volume, so it is assumed that the coordinator has enough power. For each round calculation, if *TotalPower(i->j)* + *TotalPower(j)* is smaller than *TotalPower(i)* (the total power for data transmission from node *i* to BS via a specific route), then the next hop of node *i* is pointed to node *j*. The corresponding total power is updated. The route calculation ends when the next hop array does not change. Algorithm 1 shows the route calculation algorithm. For the calculated route, the corresponding minimum transmit power can be calculated according to Equation (12):

(10) PL=Pt-RSSI

(11) $$\text{TotalPower}(i->j)=\sum_{\text{route}}{\text{Pt}}_{\text{ij}}+\sum_{\text{route}}{\text{Pr}}_{\text{ij}}$$

(12) $$Pt_{ij}(dB)=\text{Sensitivity}+PL_{ij}+P_{\text{guard}}(dB)$$

**Algorithm 1: Route calculation**
**V:** set of all the medical nodes in the networks**SET** Nexthop(node *i*) = *BS***SET** TotalPower(node *i*) = TotalPower(*i*->*BS*)**WHILE** NexthopArray changed **do** **WHILE**
*i* ∈ *V*
**do**   **WHILE**
*j* ∈ *V*, *j* ≠ *i*
**do**     **IF** TotalPower(*i*->*j*) + TotalPower(*j*)     <TotalPower(*i*) **Then**       Nexthop(*i*)=*j*       TotalPower(*i*) = TotalPower(*i*->*j*) + TotalPower(*j*)     **End if**   **End while** **End while**check if NexthopArray is changed**End while**

## 4. Performance Evaluation

A BSN with mobile sensor nodes inside the human body is simulated to evaluate the performance of the proposed protocol using MATLAB. The energy consumption of the proposed MAC protocol is compared with IEEE 802.15.6. It stated in Section 2, multi-hop communication could save more energy than single-hop communication. However, there exists control message overhead in the proposed MAC protocol. The energy consumption of the control overhead cannot be negligible. This section will analyze whether the proposed protocol could save energy or not even if there exists control message overhead.

The random mobility patterns are generated based on the assumptions given in Table 3. The GI tract is normally more than 6 meters in length and the nodal speed in the gastrointestinal tract is 0.2 mm/s [36]. Here the mobility pattern is modeled as multi-segment line around 6.3 meters inside a cube of 25 × 15 × 25 cm. The assumptions are within the range of normal adult human. Each segment follows normal distribution with the mean length of 7.4 cm and the standard deviation of 3.1 cm. The angle of each segment line to the coordinate follows a uniform distribution with the minimum value of 0° and maximum value of 360°. The mobility pattern starts at (7, 11.3, 25) and ends at (0, 0.2, 4). This mobility pattern is much more realistic and very close to the real GI tract of the human body.

The parameters used in the simulations are listed in Table 4. The mobility pattern is generated using the above-mentioned model in a 25 × 25 × 15 cm^3^ cube. Different network sizes from two sensors to six sensors are simulated. In the simulation, the channel path-loss model and energy model are the same as that in Section 2. The parameters are justified in Section 2. The circuitry power adopts 100 μW, 1 mW and 5 mW in this simulation. 405 MHz is adopted as the RF frequency. We define the frame format length as 1 s. The mini-control slot duration defined as 1 ms and the data slot duration is defined as 20 ms. The payload size for information exchange and scheduling assignment is estimated as 10 bytes and the payload size for broadcasting and information uploading is estimated as 10 × *N*, where, *N* is the number of the sensors. The sensing data packet size is defined from 512 bytes to 3 kbytes. The packet interval is from 0.2 s to 1 s. These parameters are selected as close as the real WCE. The total simulation lasts 20,000 s. For energy calculation, the total energy consumption is calculated as the summation of the energy consumption of the status tx, rx, and idle for all the sensor devices as shown in Equation (13). The energy consumptions for both control data and sensing data are calculated into the total energy consumption:

(13) $$E_{\text{total}}=\sum_{\text{sensors}}\left(E_{tx}+E_{rx}+E_{\text{idle}}\right)$$

**Figure 7.**
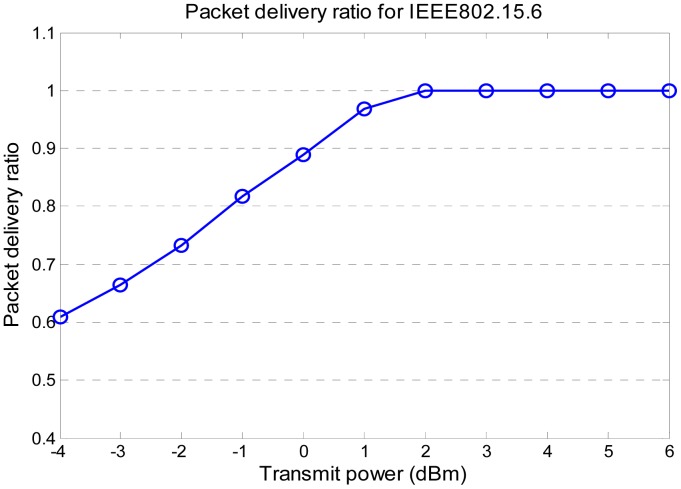
Packet delivery ratio for IEEE 802.15.6.

The IEEE 802.15.6 MAC protocol is used as reference for comparison with the proposed MAC protocol. In the simulation it adopts guaranteed time slots (GTS) in the managed access period. The sensor devices and coordinator node communicate in a point-to-point mode. In the simulation no acknowledgement is adopted, which gives a fair comparison with the proposed protocol. The data slots are equally assigned to sensor devices in advance. To make the comparison fair, we try to obtain the minimum transmission power for 802.15.6. Figure 7 shows the packet delivery ratio (PDR) *vs*. transmit power. 2 dBm is the minimum transmit power to ensure PDR of 1. For the proposed MAC protocol, the transmission power is calculated as in Equation (12). The value is calculated by the coordinator. It can guarantee that all the data are sent with adequately high transmission power to reach to the receiver.

**Figure 8.**
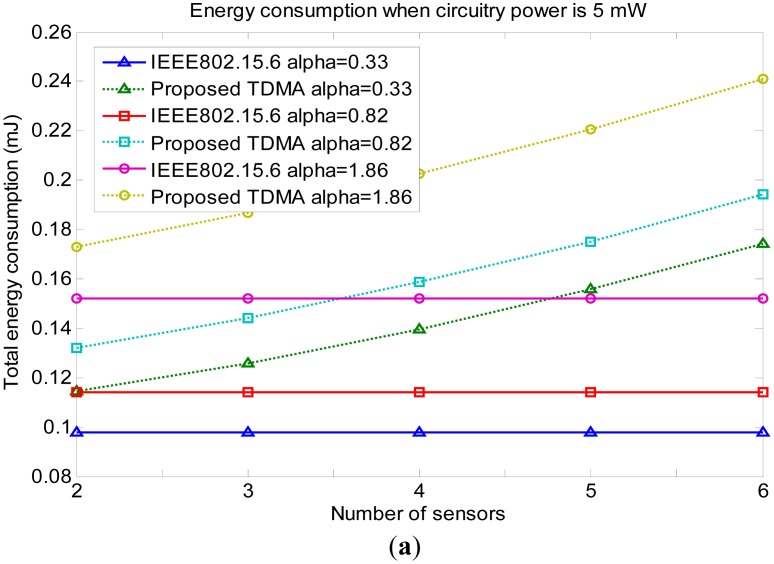

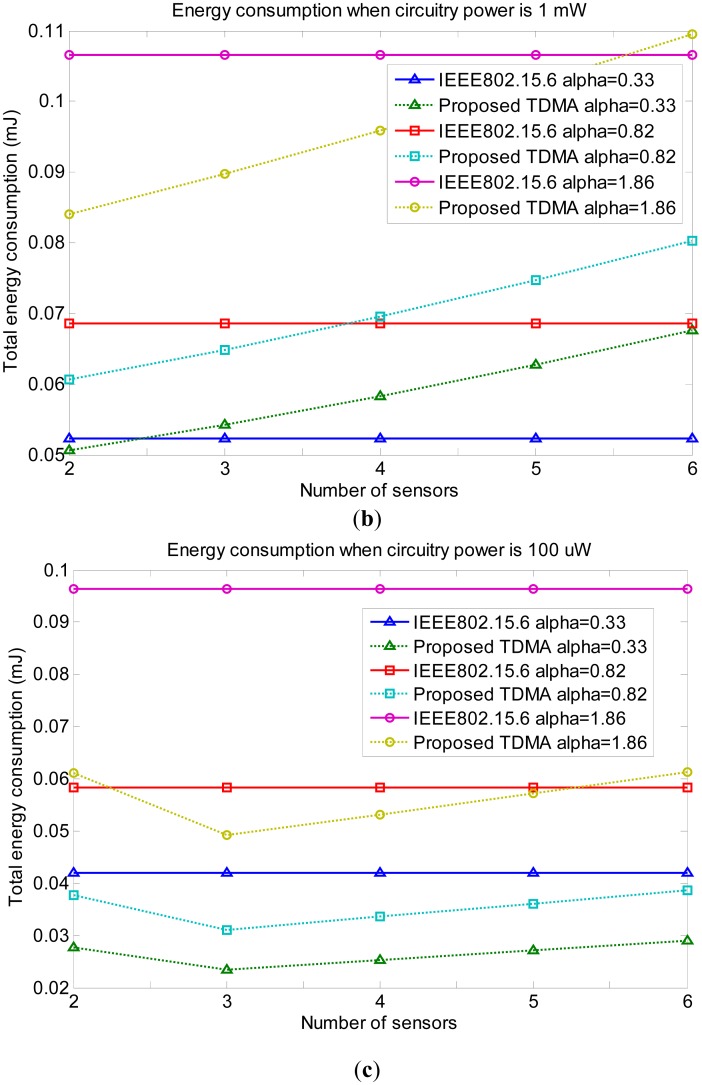
(**a**) Energy consumptions for different α circuitry power when circuitry power is 5 mW; (**b**) Energy consumptions for different α circuitry power when circuitry power is 1 mW; (**c**) Energy consumptions for different α circuitry power when the circuitry power is 100 μW.

The energy consumptions of the proposed protocol and IEEE 802.15.6 are compared for different circuitry powers (5 mW, 1 mW, 100 μW) and different *α* values (0.33, 082, 1.86; the selection of these values is explained in Section 2) in Figure 8. All the simulations obtain the PDR of 1. It can be seen that the larger *α* is, the bigger the total energy consumption is. This has been explained in Section 2. In Figure 8a, when the circuitry power is equal to 5 mW, IEEE 802.15.6 consumes less energy than the proposed protocol. This is because multi-hop communication would cause bigger energy consumption due to the repeated circuitry energy consumption. The calculated route would be 1-hop. Since the proposed protocol has relatively large overhead, the consumed energy is higher than IEEE 802.15.6. In Figure 8b, when the circuitry power is equal to 1 mW, the energy consumption of the proposed protocol and IEEE 802.15.6 are comparable. If there are two sensors in the network, the proposed protocol consumes less energy than IEEE 802.15.6. As the network size increases, the energy increases linearly. This indicates the calculated routing is 2-hop. Even if more sensors are put into the network, after the information exchange, the coordinator still calculates 2-hop as the best route. In Figure 8c, the circuitry power is equal to 100 μW. The energy consumption of the proposed TDMA is smaller than 802.15.6. When the circuitry power becomes small, multi-hop communication will not incur large energy increases from the circuitry power, but the decrease of transmission power is obvious, so the energy consumption of the proposed MAC is less than IEEE 802.15.6. A 3-sensor network achieves the lowest energy consumption. If there are two sensors in the network, the route is 2-hop. If there are three sensors in the network, the energy consumption becomes lower. This indicates 3-hop is the calculated route. The transmission power decreases. The circuitry power is very small, so one more relay does not consume too much energy. As the network size increases, the energy increases linearly. This indicates the calculated routing is still 3-hop. For the network size bigger than three, the extra sensors only participate in the control section. They consume energy but do not participate in sensing data transmission.

In summary, if the circuitry power is small enough (smaller than 1 mW which is already achieved in lab designs), the proposed MAC protocol would save energy compared with IEEE 802.15.6 for mobile swallowable BSNs.

## 5. Conclusions

Energy efficiency is one of the major concerns of BSNs. Many energy efficient MAC protocols have been proposed. This paper considers adopting multi-hop communication in mobile swallowable BSNs for the first time. The paper evaluates multi-hop communication though the human body based on the IEEE 802.15.6 body channel model. The results showed that multi-hop communication saves energy consumption compared with single-hop communication when the circuitry power is small enough. Based on the result, the TDMA schedules for medium access are given in detail. The coordinator collects the neighboring information from sensors and calculates the routing and transmission power for each sensor. The sensors follow the schedules to finish data transmission. The proposed protocol avoids the idle listening, overhearing problem and most of the processing work is shifted to the coordinator. Simulation results showed the proposed TDMA protocol gives better performance than IEEE 802.15.6 in terms of energy consumption when the circuitry power becomes low. The main reason is that multi-hop communication makes the transmit power smaller and the corresponding adaptive power control. As the circuitry power becomes smaller due to new technologies, the proposed TDMA MAC protocol could be a good reference for energy efficient solution of BSN MAC standard. The future work would focus on slot assignment algorithm and hardware experiments.

## Figures and Tables

**Table 1.** Parameters used in path-loss estimation.

Implant to Implant	*PL*(*d*_0_)(dB)	*n*	σ*_S_* (dB)

Deep Tissue	35.04	6.26	8.18
Near Surface	40.94	4.99	9.05

Implant to Body Surface	*PL*(*d*_0_)(dB)	*n*	σ*_S_* (dB)

Deep Tissue	47.14	4.26	7.85
Near Surface	49.81	4.22	6.81

**Table 2.** Simulation parameters for the evaluation of multi-hop communication.

**Parameters**	**Value**
Scenarios	
Area	40 cm straight line
Deployment	Uniform distribution
Number of hops	1–5

Path-loss model	IEEE802.15.6

Energy model	
Sensitivity	−84 dBm
Transmit power	Sensitivity + path-loss
Alpha	1
Circuitry power	
Transmit mode	100 μW–15 mW
Receive mode	100 μW–15 mW

Application traffic	
Data rate	2 packets/sec
Packet size	200 bytes

Simulation duration	30 s

**Table 3.** Assumptions for random mobility pattern generation.

**Parameters**	**Value**	**Parameters**	**Value**
Maximum angle	360	Mean of distance	7.361 cm
Minimum angle	0	Standard deviation	3.1229 cm
Length of small intestine	∼630 cm	Border	25 × 15 × 25 cm
Starting point	(7,11.3,25)	Destination point	(0, 0.231, 4)

**Table 4.** Parameters used in simulation.

**Parameters**	**Value**
Scenarios	
Area	25 × 25 × 15 cm^3^
Mobility pattern	Generated GI tract model
Number of sensors	2–6

Path-loss model	IEEE802.15.6
Channel frequency	405 MHz

Energy model	
Sensitivity	−84 dBm
Transmit power	Adaptive
Alpha	0.33, 0.82, 1.86
Circuitry power	
Transmit mode	100 μW, 1 mW, 5 mW
Receive mode	100 μW, 1 mW, 5 mW

Frame length	1 s
Control slot duration	1 millisecond
Data slot duration	20 milliseconds
Routing update frequency	1

Application traffic	
Data packet interval	0.2 s–1 s
Data packet size	512 bytes – 3 kbytes
Single control packet size	10 bytes

Simulation duration	20,000 s
